# Conventional and fluorescent based semen quality assessment in Karan Fries bulls

**DOI:** 10.14202/vetworld.2015.1243-1246

**Published:** 2015-10-28

**Authors:** A. Panmei, A. K. Gupta, P. R. Shivahre, M. Bhakat, A. Upadhyay

**Affiliations:** Division of Dairy Cattle Breeding, Indian Council of Agricultural Research, National Dairy Research Institute, Karnal, Haryana, India

**Keywords:** conventional method, fluorescent technique, Karan Fries, semen quality

## Abstract

**Aim:**

The present study was carried out on semen ejaculates of 15 Karan Fries (KF) bulls maintained at Artificial Breeding Research Centre, National Dairy Research Institute, Karnal, India with an objective to evaluate the relationship between the conventional and fluorescent based semen quality analysis of the bulls.

**Materials and Methods:**

A total of 96 ejaculates were collected from 15 KF (Holstein Friesian [HF] crossbred) bulls. Semen were evaluated for color, volume, mass activity (MA) and percentage of individual motility (IM), sperm concentration, percent live spermatozoa, hypo-osmotic swelling test and acrosome integrity, chromatin integrity, sperm viability, and membrane integrity. Data were analyzed using SPSS software package for descriptive analysis. The correlation between rankings of sires based on conventional and fluorescent semen parameters were calculated by Spearman’s rank correlation coefficient.

**Results:**

The average ejaculates volume (ml), sperm concentration (10^6^/ml), MA, IM (%), live (%), morphological abnormalities (%), host (%), acrosome integrity (%), chromomycin A3 (CMA3) (%), SYBR-PI (%), and fluorescent isothiocyanate-peanut agglutinin (FITC-PNA) (%) were 4.57±0.36, 1162.98±97.93, 2.95±0.09, 60.8±1.22, 71.41±2.10, 9.31±1.15, 65.5±1.81, 86.6±1.59, 3.53±0.43, 65.39±2.23 and 74.47±2.53, respectively. Rank correlations were found to be significant for SYBR-PI and FITC-PNA with most of the parameters evaluated by conventional methods. Overall, among conventional criteria, IM revealed ranking of bulls almost similar to that of fluorescent criteria.

**Conclusion:**

Overview of our results indicated that, among conventional criteria, MA and IM revealed ranking of bulls almost similar to that of fluorescent criteria.

## Introduction

Analysis of seminal parameters help in providing important clinical information regarding spermatogenesis, the functional competence of spermatozoa and also the secretory pattern of accessory genital glands [1,2]. To assess the fertility status of the male, different tests have been developed and are routinely used for evaluation of the quality of a semen sample. The likely causes of variability in semen quality measurement are human bias, variation in personnel training, and use of different methods to evaluate seminal quality [3,4]. Conventionally, the semen is evaluated on the basis of motility, morphology and viability [5]. Moreover, conventional measurements are prone to extreme inter-ejaculate variation, even when the laboratory methodology has been standardized.

In recent years, more attention has been given to evaluating sperm by fluorescent techniques which offer many advantages over conventional techniques of sperm quality. These new methods help in evaluating the ultra-structural damage, particularly to organelles [6,7]. However, fluorescent technique implies a high cost, need experienced personals, time consuming, less repeatable technique, etc., and is not suitable or inconvenient to use in field conditions for routine semen analysis. For these reasons, semen evaluation process having a high correlation between the two different techniques, which can offer high accurate and reliable results, is needed.

The objectives of this present study was to find out the possible correlation between conventional and fluorescent methods of evaluating bull spermatozoa in Karan Fries (KF) bulls in an effort to determine which types of assays are more important to provide the greatest information regarding the quality of a semen sample, enabling relatively quick and inexpensive laboratory assays with good results in general.

## Materials and Methods

### Ethical approval

The present study was approved by Institutional Animal Ethics Committee of National Dairy Research Institute.

### Collection of semen samples

Semen was collected once or twice a week where two successive ejaculates (15-30 min gap) were taken in each collection. The total of 96 ejaculates were collected from 15 KF (Holstein Friesian [HF] crossbred) bulls (4-6 ejaculates each bull) maintained at Artificial Breeding Research Centre, National Dairy Research Institute (NDRI), Karnal, to evaluate the *in vitro* fertility based on conventional and fluorescent seminal characteristics. Semen was evaluated for color, volume, mass activity (MA), and percentage of individual motility (IM). Sperm concentration was estimated by hemocytometer using Neubauer cell counting chamber. Semen samples are diluted at 1:200 with diluting fluid (1% NaCl and 1% Formaldehyde) and spermatozoa were counted in 5 secondary squares that are meant for counting red blood cells. Live sperm % was assessed by eosine-nigrosin staining technique where, live spermatozoa remain unstained and appear to be white while dead sperm shows red or pink, hypo-osmotic swelling test using hypo-osmotic solutions of 150 mOsmol-1 and acrosome integrity by giemsa stain. Chromatin integrity of each sperm was quantified by epifluorescent microscope (Olympus) after staining with CMA3 fluorescence stain as described by Bianchi *et al*. [8]. To validate the quantitative assessment of the sperm viability and membrane integrity, SYBR-PI was used as described by Januskauskas *et al*. [9]. Fluorescent isothiocyanate-peanut agglutinin (FITC-PNA) stain was used for assessment of acrosome integrity as described by Roth *et al*. [10].

### Statistical analysis

Data were analyzed using SPSS software package for descriptive analysis. The repeatability of various seminal attributes of KF bulls was estimated as intraclass correlation from Analysis of Variance [11] using records of the same animal in successive collections. The standard error of repeatability was estimated by using the formula as given by Swiger *et al*. [12]. By using the repeatability estimates, expected producing ability (EPA) of the semen quality was computed for different semen parameters for each of the KF breeding bulls. EPA of the breeding bulls with a different number of records is compared within a herd as estimated by Lasley [13]. The correlation between rankings of sires based on conventional and fluorescent semen parameters were calculated by Spearman’s rank correlation coefficient.

## Results and Discussion

The mean±standard error and repeatability estimates of the semen quality parameters of KF bulls by conventional and fluorescent technique were presented in Table-1. The average ejaculate volume was comparable with the values reported by Ulfina and Raina [14], and Bhakat [15] in HF crosses while lower values than our present finding was reported by Mathur *et al*. [16]. The variation in semen volume may be due to different genetic inheritance of the cross bred bulls. The average values of MA and IM were comparable with the findings of Panwar and Nagpaul [17] and Garge and Pandit [18], respectively. Finding of the average concentration of spermatozoa for KF bulls was in agreement with Singh and Pangawkar [19]. However, higher values were reported by Mathur *et al*. [16]. The differences in the reports may possibly be due to differences in seasons of the collection, age of bulls, genetic difference, and ejaculation frequency. The overall average values of percent live spermatozoa and morphological abnormal spermatozoa in KF bulls was reported to be comparable with the findings reported by Raja and Rao [20] and Sharma *et al*. [21], respectively. For the fluorescent based technique of different seminal attributes in KF bulls, the values for CMA3 positive spermatozoa was higher than that estimated by Rajak [22] (2.5±0.85%) and Singh [23] (1.93±0.90%). The results of percent membrane intact spermatozoa (SYBR-PI) was higher than that reported by Vijetha [24] (52.61±1.43) but lower than that reported by Singh [23] (67.08±0.97%). A perusal of literature on overall mean of live acrosome reacted spermatozoa in neat semen revealed higher values than that reported by Sandeep [25] (40.00±0.75).

**Table-1:** Mean±SE and repeatability estimates of semen quality parameters of KF bulls (total number of ejaculates=96) based on conventional and fluorescent technique.

Parameters	Mean±SE	Repeatability±SE
Volume (ml)	4.75±0.36	0.405±0.121
Sperm concentration (10^6^/ml)	1162.98±97.93	0.397±0.121
MA (0-5 scale)	2.95±0.09	0.643±0.102
IM (%)	60.8±1.22	0.643±0.102
Live cells (%)	71.41±2.10	0.614±0.107
Abnormal sperm (%)	9.31±1.15	0.709±0.089
Host positive cells (%)	65.5±1.81	0.459±0.120
Acrosomal integrity (%)	86.6±1.59	0.402±0.121
CMA3 (%)	3.53±0.432	0.657±0.109
SYBR-PI (%)	65.39±2.229	0.601±0.105
FITC-PNA (%)	74.47±2.527	0.452±0.115

SE=Standard error, KF=Karan Fries, FITC-PNA=Fluorescent isothiocyanate-peanut agglutinin, MA=Mass activity, IM=Individual motility

The estimates of repeatability were significant and high for most of semen characteristics in KF and varied from 0.402 for acrosome integrity to 0.709 for morphologically abnormal spermatozoa. Boujenane and Boussaq [26] reported repeatability estimate to be 0.157 for mass motility and 0.411 for ejaculate volume. In addition, Taylor *et al*. [27] reported that repeatability estimates for volume, concentration, and number of spermatozoa per ejaculate were 0.26, 0.23, and 0.37, respectively. The higher repeatability estimates indicate that bulls tend to have the same performance for traits with high estimates. Mathevon *et al*. [28] found that repeatability estimates were higher for mature bulls (from 0.51 to 0.64) than for young bulls (0.41-0.53) owing to their more stabilized semen production. Karoui *et al*. [29] reported repeatability estimates of mass motility and IM as 0.37 and 0.35, respectively, in HF.

Association among the semen quality parameters of ranked KF bulls based on conventional and fluorescent techniques were estimated by rank correlation and presented in Table-2. Each KF breeding bulls were ranked based on their EPA for semen quality obtained for different semen parameters in conventional techniques and fluorescent techniques. The rank correlation between the chromatin integrity and MA as well as IM were statistically significant (p<0.05) indicating that ranked bulls based on chromatin integrity were found to be positively correlated with the bulls which were ranked based on MA and IM. Previous studies reported by Giwercman *et al*. [30] and Sills *et al*. [31] showed negative correlation between DNA damage and DNA fragmentation index and other conventional semen parameters, such as motility, morphology, and concentration. The rank correlations of the ranks assigned to different breeding bulls evaluated by fluorescent based membrane integrity and viability (SYBR-PI) were statistically significant with those assigned on the basis of conventional semen assessment criteria of MA, IM, percent live spermatozoa, and HOST values. The rank correlation between acrosome integrity (FITC-PNA) and acrosome integrity (conventional technique) was statistically significant (p<0.01). The rank correlations between FITC-PNA and MA and IM test were significant (p<0.05). Overall, the results showed that *in-vitro* evaluation of breeding bulls by fluorescent based CMA3, SYBR-P1, and FITC-PNA test were highly correlated with MA and IM tests. SYBR-PI and FITC-PNA tests were also highly correlated with percent live spermatozoa and acrosomal integrity of conventional based semen analysis, respectively.

**Table-2:** Rank correlation among conventional and fluorescent seminal attributes.

Parameters	MA	IM	Percent live	Morphological abnormality	Host	Acrosomal integrity
Chromatin integrity (CMA3)	0.613*	0.607*	0.445	0.464	0.345	0.420
Membrane integrity and viability (SYBR-PI)	0.614*	0.630*	0.854**	0.277	0.614*	0.407
Acrosomal integrity (FITC-PNA)	0.525*	0.541*	0.389	0.284	0.482	0.843**

* t(p<0.05),

** t(p<0.01), FITC-PNA=Fluorescent isothiocyanate-peanut agglutinin, MA=Mass activity, IM=Individual motility

## Conclusion

Routine semen analysis is helpful to estimate the *in-vitro* fertility of a bull, but it is not always reliable. Therefore, advancements on bull semen analysis by evaluation of sperm function in depth as to estimate the sperm nucleus chromatin integrity, sperm plasma membrane, and acrosome integrity have been included to bull semen assessment to provide additional information that may be useful in predicting the fertilization potential of spermatozoa. The rank correlations from our results indicated that MA and IM are important conventional tests which revealed more similar ranking of the bulls as that of the fluorescent tests.

## Authors’ Contributions

AKG designed the work. AP conducted the study and analyzed the data. MB helped in the compilation of data. PRS and AU helped in writing and revision of the manuscript. All authors read and approved the final manuscript.
